# Supplementary figures and images for: Biological effects of corticosteroids on pneumococcal pneumonia in Mice—translational significance
Source: Crit Care. 2024 May 29;28:185. doi: 10.1186/s13054-024-04956-6 (PMC11134653; doi:10.1186/s13054-024-04956-6)

Figure S1.

A

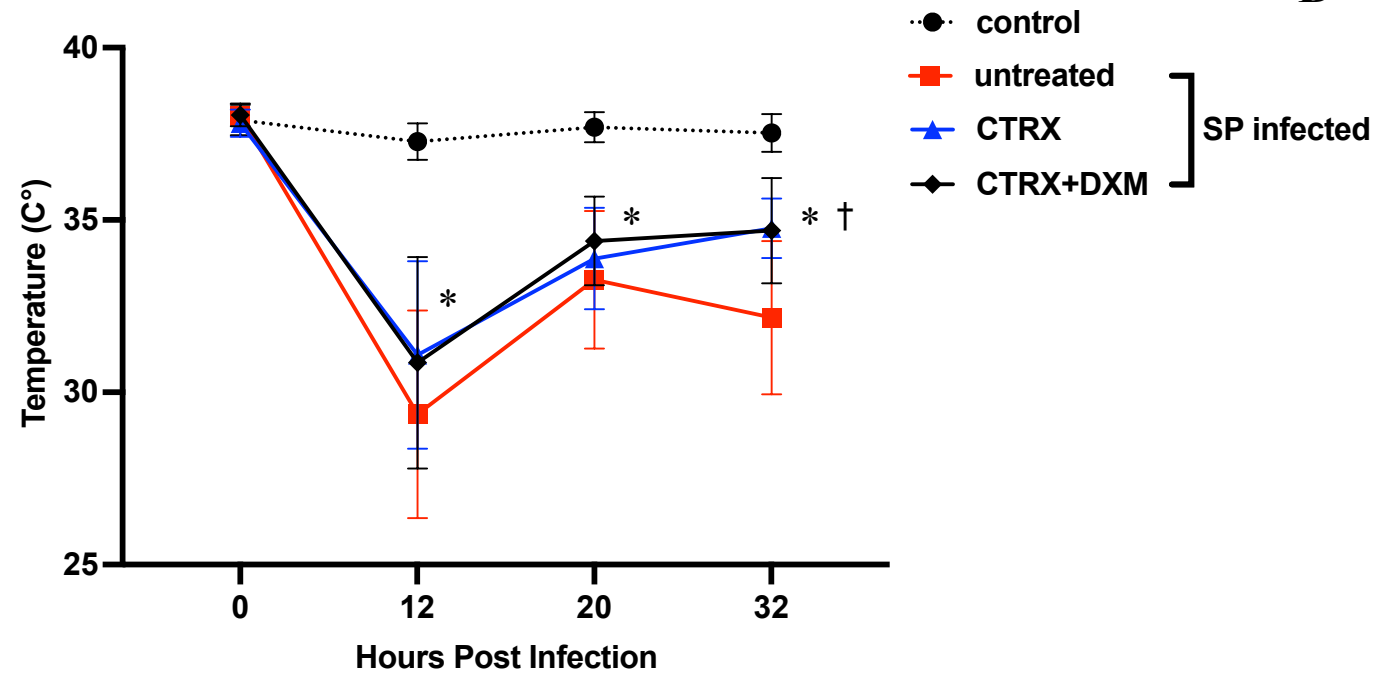

B

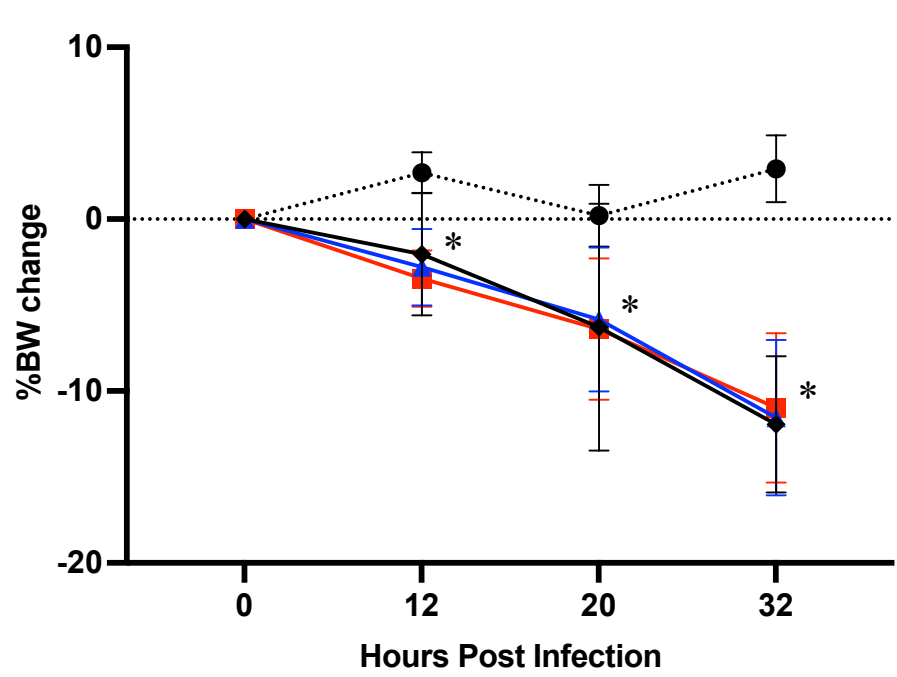

Figure S2.

A

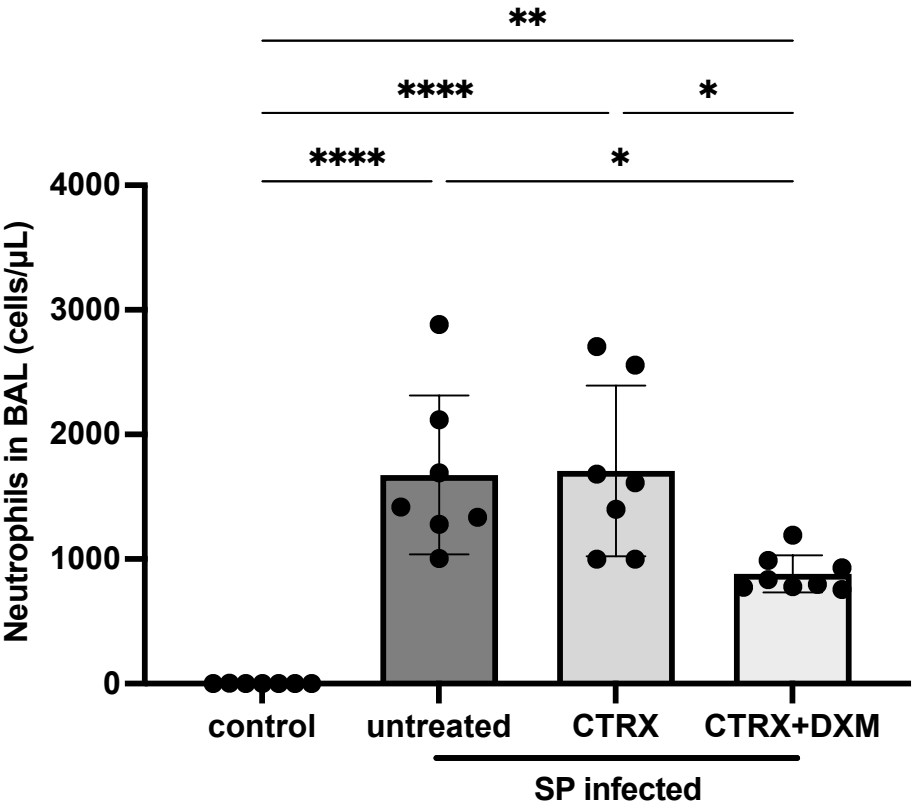

B

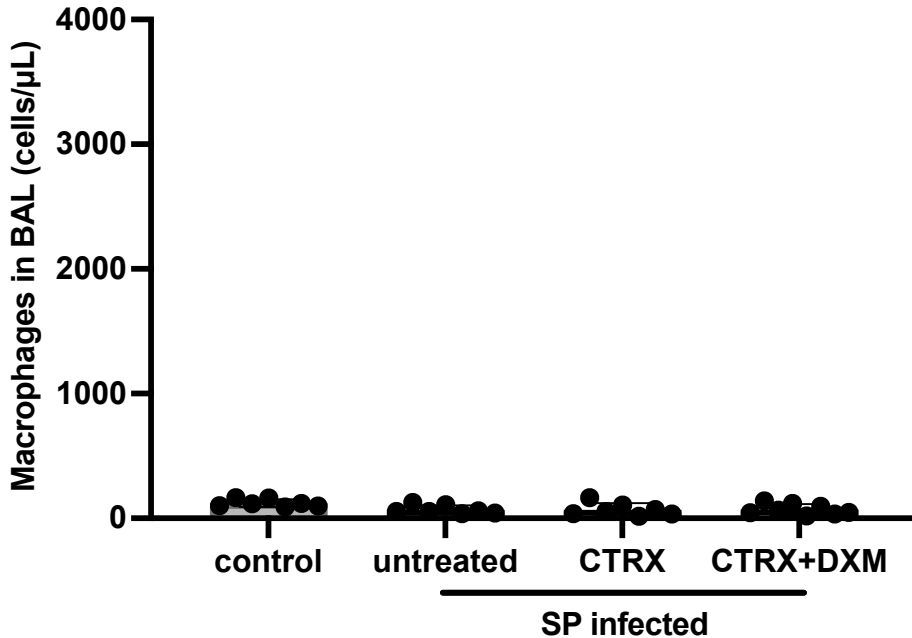

Figure S3.

A

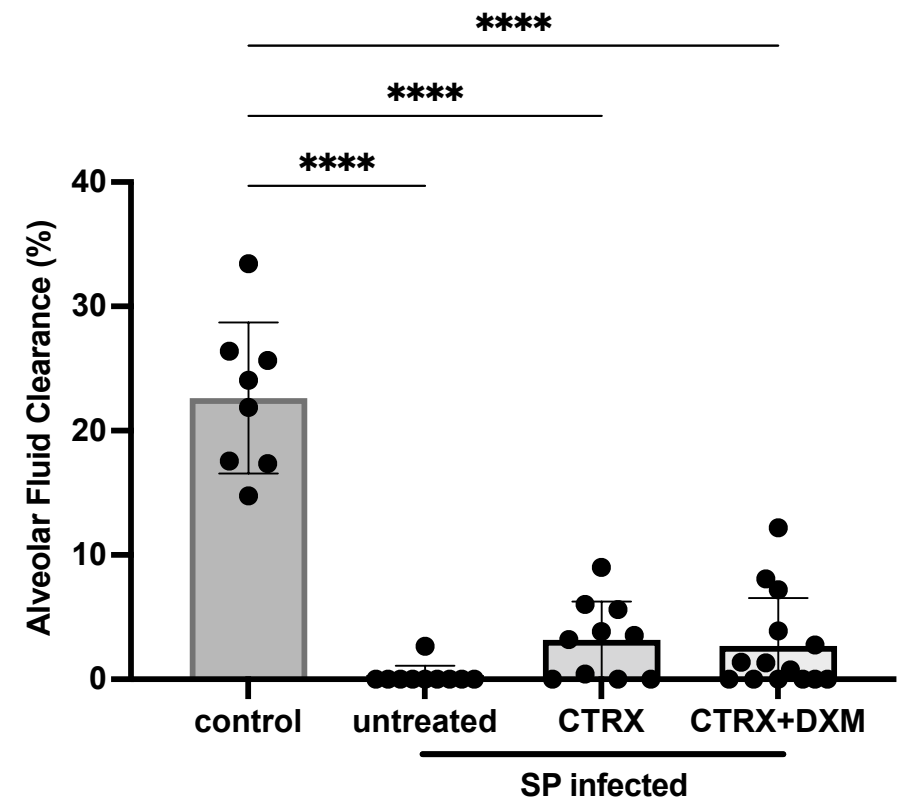

B

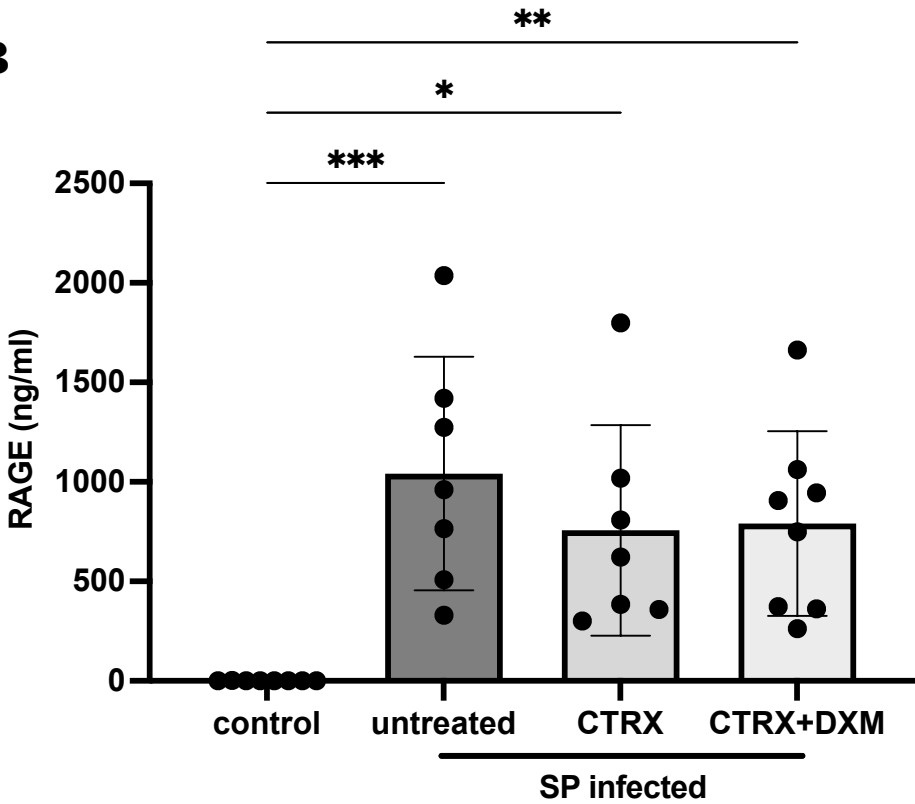

Figure S4.

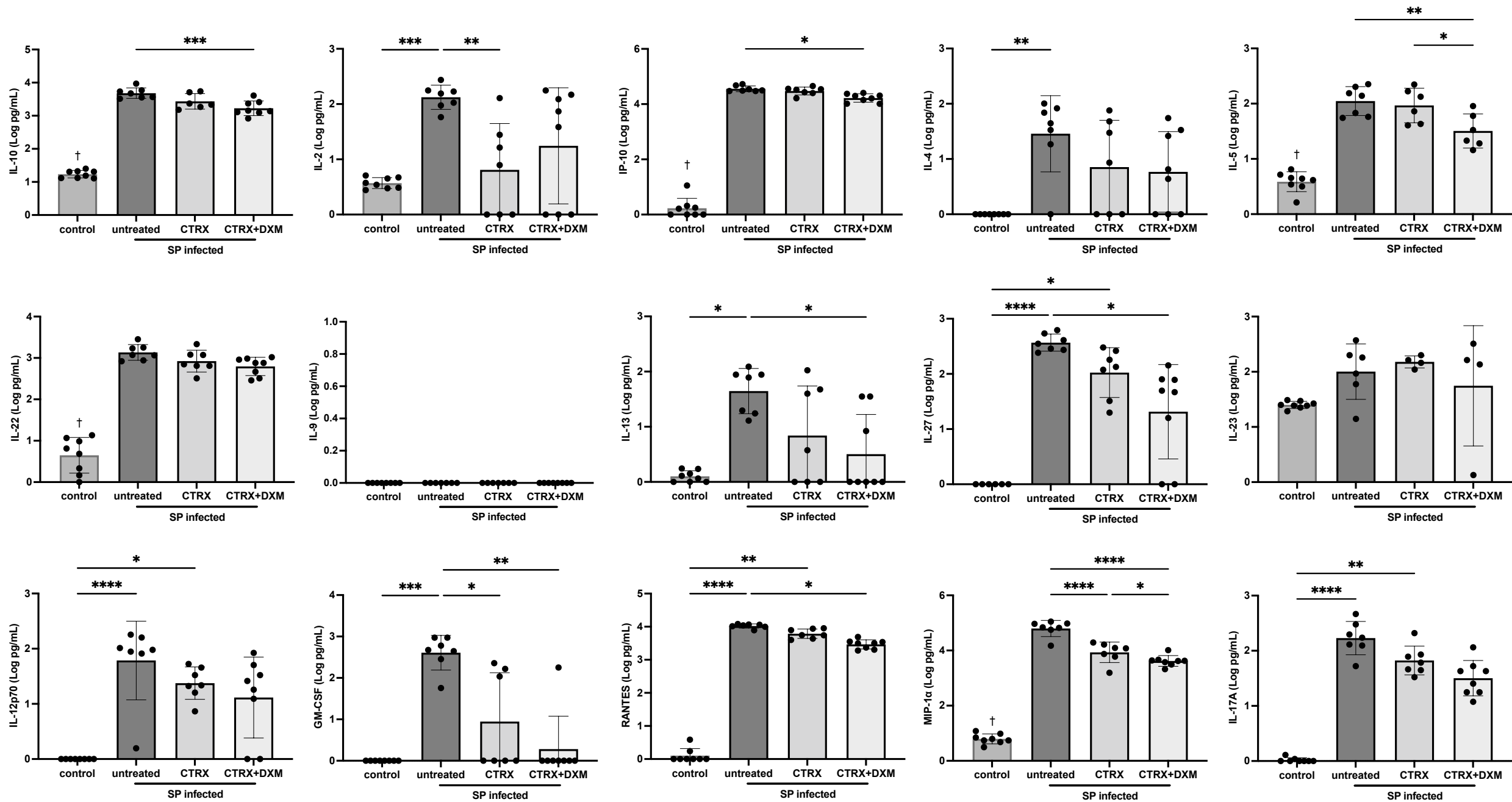

Figure S4. (continued)

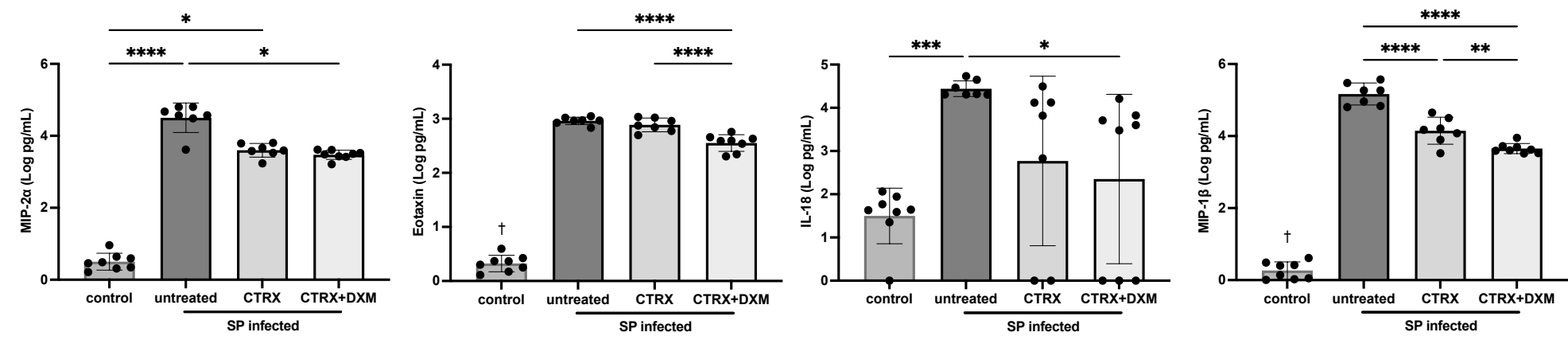

Figure S5.

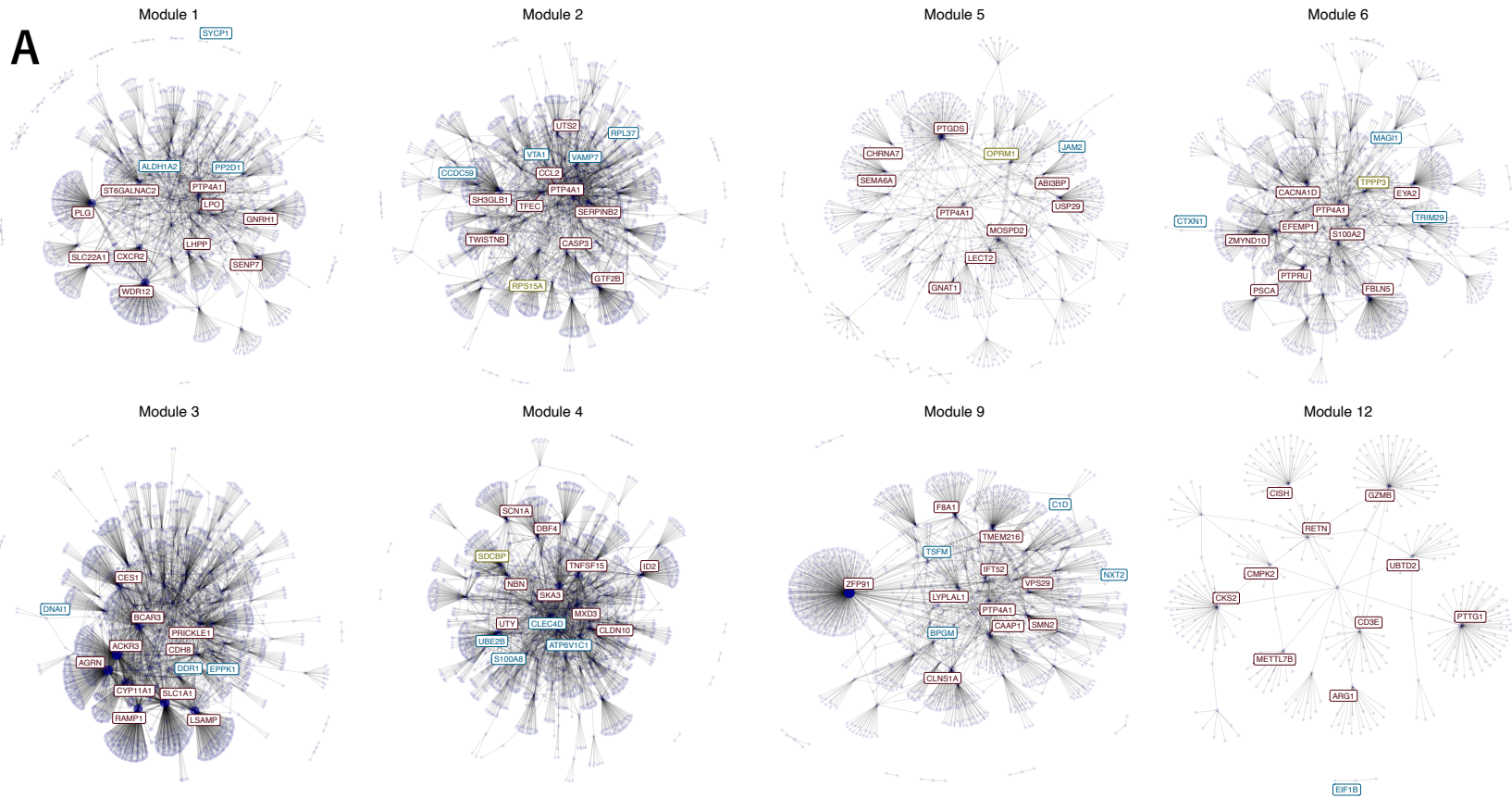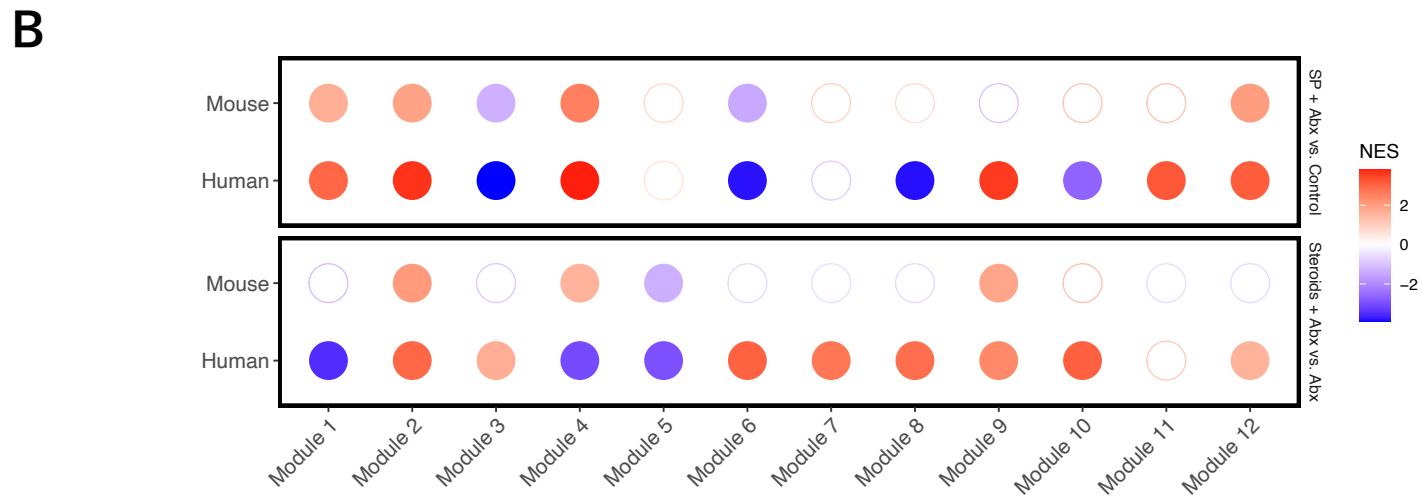

Figure S6.

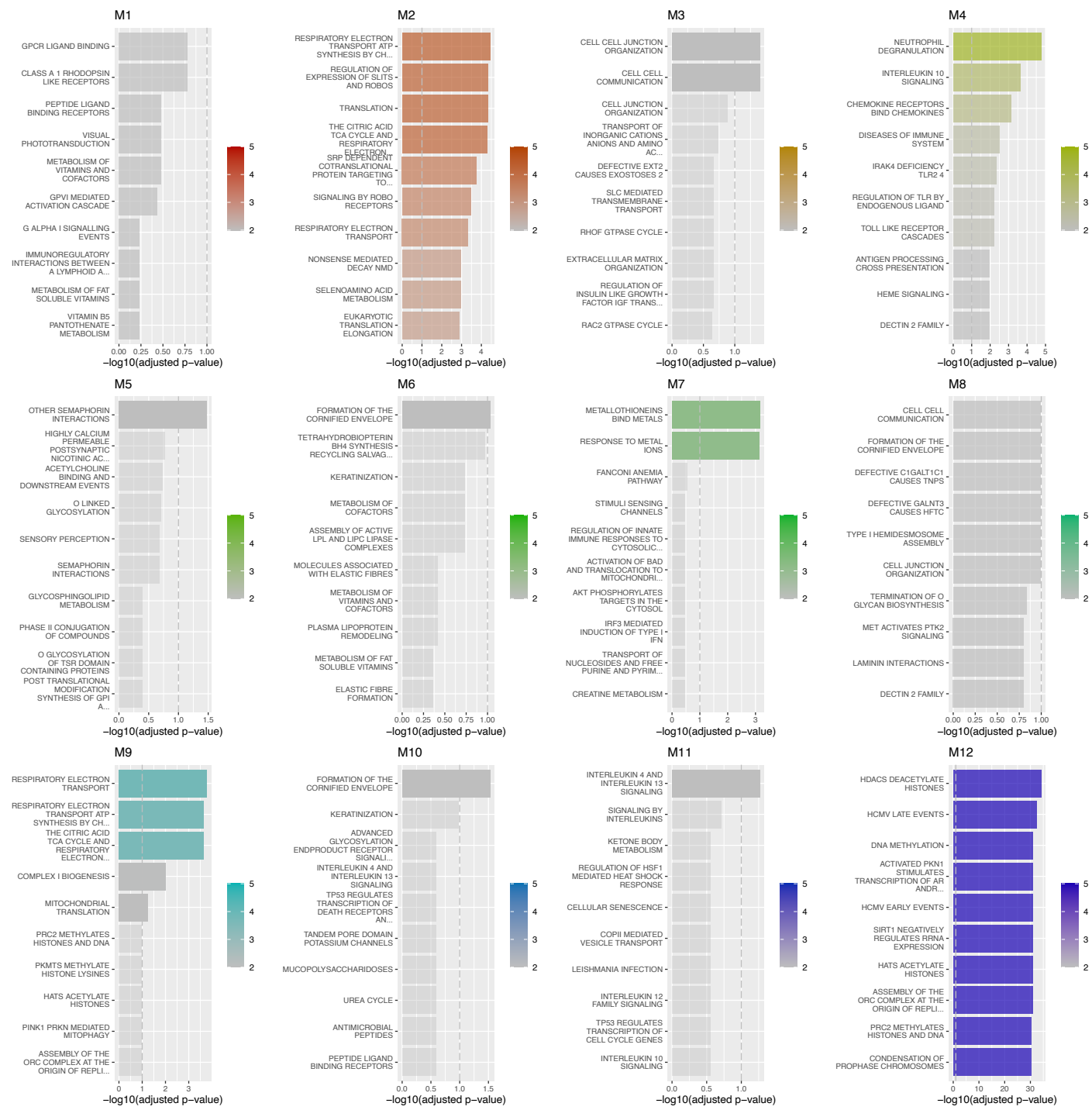

Supplement: Supplementary file 1 — Additional file 1. Supplemental Figures. [file 13054_2024_4956_MOESM1_ESM.pdf]
