# Supplementary material for: Biological effects of corticosteroids on pneumococcal pneumonia in Mice—translational significance
Source: Crit Care. 2024 May 29;28:185. doi: 10.1186/s13054-024-04956-6 (PMC11134653; doi:10.1186/s13054-024-04956-6)
Supplement: Supplementary file 2 — Additional file 2. Supplemental File. [file 13054_2024_4956_MOESM2_ESM.docx]

**SUPPLEMENTARY Appendix**

**List of contents**

**APPENDIX 1- Methodology**………………………………………………………...2

**APPENDIX 2- Supplemental results**………………………………………………..9

**APPENDIX 3- Supplemental figure legend**………………………………………..10

**APPENDIX 4- Supplemental table**………………………………………………....13

**REFERENCES**…………………………………………..……………………..……14

**Apendix-1. METHODOLOGY**

***Observational patient cohort:*** The study was approved by the UCSF Institutional Review Board (17-24056). Patients were enrolled within 72 hours of ICU admission and all tracheal aspirate samples were collected on the day of enrollment. If a patient was able to consent or had a surrogate available, a study physician or research coordinator obtained written informed consent at the time of enrollment. For subjects who could not consent at enrollment, the UCSF IRB granted an initial waiver of informed consent for biospecimen and clinical data collection, as previously described [1]. The UCSF IRB also approved a full waiver of consent for patients who died prior to regaining an ability provide informed consent, but restricted data sharing for samples collected from these patients. Because some of the subjects included in the present study were enrolled under a waiver of consent, our IRB protocol prohibits the public release of raw sequencing data, which may contain personally identifiable genetic information. Seven of the *S. pneumoniae* subjects were previously included in a study of metagenomic sequencing in mechanically ventilated patients [2], and the five control subjects were previously included in an analysis of patients with COVID ARDS [1]. Gene count data for all subjects in this study are included in the supplementary material.

***Animals:*** Adult 10- to 12-week-old female C57BL/6 mice purchased from the National Cancer Institute (Frederick, MD) were used for all experiments. All work was conducted under a protocol approved by the University of California, San Francisco Institutional Animal Care and Use Committee (IACUC) (No.AN189182).

***Experimental Protocol:*** Mice were randomly divided into four groups as follows:

(1) Healthy normal control

(2) *S. pneumoniae*

(3) *S. pneumoniae* + ceftriaxone

(4) *S. pneumoniae* + ceftriaxone + dexamethasone

*S. pneumoniae* serotype 19F [American Type Culture Collection (ATCC) 49619, Manassas, VA] was grown in brain-heart broth (Becton Dickinson 237500, Sparks, MD) and harvested at the mid-log phase [optical density (OD) 0.50 at 600 nm]. To improve reliability across experiments, all cultures were derived from aliquots of a single bacterial expansion frozen at -80°C in 30% glycerol. Once OD 0.5 was reached, the bacterial culture was spun at 3,000 g and 4°C for 10 min and resuspended in sterile PBS. The bacterial solution was adjusted to achieve 2 x 10^9^ colony-forming units (CFU)/ml. Mice were anesthetized deeply with isoflurane (3%) and inoculated intranasally with 10^8^ CFU (50µl) of live *S. pneumoniae*. Mice received 10 mg/kg of dexamethasone or vehicle control in conjunction with 150 mg/kg of ceftriaxone or vehicle control intraperitoneally 20 and 32 hours after inoculation. Ceftriaxone was reconstituted in normal saline. In some experiments, body temperature, body weight, and oxygen saturation by pulse oximetry (SpO_2_) were measured 12, 20, and 32 hours after inoculation. Pulse oximetry was measured using the MouseOx+ cervical collar system (Starr Life Sciences, Oakmont, PA) after shaving the neck of mice. We monitored mice for 5 minutes per time point, and the mean SpO_2_ for 10 seconds when mice were not active was calculated. All mice were closely monitored throughout the experiments and were sacrificed 36 hours after infection, or earlier if the mice were moribund and it was considered inhumane to continue the experiment (weight loss >20% of starting body weight, severe respiratory distress, inability to ambulate, persistently palpable hypothermia).

***Lung Injury Endpoints:***

***Excess lung water (measure of pulmonary edema).*** Mice underwent overdose of ketamine (500mg/kg i.v.) and xylazine (50mg/kg i.v.), bilateral thoracotomy, and exsanguination by right ventricular puncture. In some mice, lungs were removed and homogenized in 1 mL PBS, and samples of blood, lung homogenate, and homogenate supernatant were weighed before and after desiccation. Systemic hemoglobin and hematocrit were measured with a GENESIS^TM^ (Oxford Science Inc., Oxford, CT). Another fraction of homogenate was assayed for hemoglobin concentration, and the blood volume of the lung was calculated, permitting assessment of the excess extravascular lung water (ELW, i.e., pulmonary edema in the interstitial and alveolar spaces above the level in normal mice of the same size) [3, 4].

***Bronchoalveolar lavage (BAL).*** In other mice, after exsanguination, the trachea was cannulated, and the lungs were lavaged twice with 250 µL of PBS. Bronchoalveolar lavage (BAL) protein was measured with the BCA Protein Assay (Thermo Fisher Scientific, Waltham, MA). BAL cell count was measured with a Coulter counter. Cytospin preparations of BAL fluid were made and stained with Hema 3 solution (Thermo Fisher Scientific, Waltham, MA), and 200 cells/ mouse were analyzed at x100 magnification and classified as neutrophils, lymphocytes, or macrophages. Postmortem bacterial titers of BAL were measured by serial dilution and plaque counting on sheep blood agar plates. Log transformation was used for airspace bacterial load given the large variability within groups.

***Histology.*** For lung pathology, the left lungs were fixed by intratracheal installation of 1 mL of 4% paraformaldehyde followed by overnight fixation, dehydration, paraffin embedding, and staining of 4µm sections with hematoxylin-eosin. The histopathological analyses of the left and right lungs were done blindly by an investigator (XF). Lung injury was assessed based on the recommended scoring system partly modified from the report of the American Thoracic Society [5]. The scale used was from 0 to 3 for the following criteria: A) neutrophils in the alveolar space (0: none, 1: 1-10, 2: 10-20, 3: >20), and from 0 to 2 for each of the following criteria: B) neutrophils in the interstitium (0: none, 1: 1-5, 2: >5), C) proteinaceous debris filling the airspaces (0: none, 1: 1, 2: >1), and D) the extent of the septal thickening (0: <2x, 1: 2x-4x, 2: >4x). The final score was the result of the following calculation: Histology score = [(20 x A) + (14 x B) + (7 x C) + (2 x D)] / (number of fields x 100). Four fields per lung were assessed.

***Measurement of Protein Biomarkers.*** The receptor for advanced glycation end products (RAGE) as an alveolar epithelial cell injury biomarker [6] was measured using the Mouse Quantikine ELISA kit (R&D Systems, Minneapolis, MN). BAL inflammatory cytokines and chemokines were measured using a FLEXMAP 3D^TM^ (Luminex) with a 26 plex kit (Cytokine & Chemokine 26-Plex Mouse ProcartaPlex, Thermo Fisher Scientific). GM-CSF, IFN-γ, IL-1β, IL-2, IL-4, IL-5, IL-6, IL-12p70, IL-13, IL-18, TNF-α, IL-9, IL-10, IL-17A, IL-22, IL-23, IL-27, IP-10, Eotaxin, KC (CXCL-1), MCP-1, MCP-3, MIP-1α, MIP-1β, MIP-2, and RANTES were measured. For the analysis, values that were undetectable were treated as having a value of zero. Log transformation was used for cytokines and chemokines given the large variability within groups. Values below the level of detection were treated as zero post-log transformation rather than -1 [4].

***Alveolar Fluid Clearance.*** Alveolar fluid clearance was measured according to previously published methods [7, 8]. Mice underwent overdose of ketamine (500mg/kg i.v.) and xylazine (50mg/kg i.v.). Tracheostomy was done with a 20-gauge plastic catheter (Luer-Stub Adapter, Becton Dickinson, Sparks, MD). The lungs were inflated with 7 cmH_2_O continuous positive airway pressure with 100% oxygen throughout the experiment. Body temperature was maintained at 37-38℃ by heat pad (Physitemp TCAT-2LV Animal Temperature Controller, Physitemp, Clifton, NJ) and an infrared lamp placed 30 cm above the body. The lamp was cycled on and off to maintain the core temperature at 37-38℃. 400 μL of 5% bovine serum albumin (BSA, Sigma-Aldrich, St Louis, MO) plus 0.1% albumin-fluorescein isothiocyanate conjugate (FITC-albumin, Sigma-Aldrich, St Louis, MO) in PBS was instilled. Five and 35 minutes after instillation, fluid was aspirated. Fluorescence (excitation at 490 nm) in the initial (Fi) and final (Ff) sample was measured at an emission wavelength of 525 nm and used to estimate alveolar fluid clearance (percent per 30 minutes), calculated as (1-Fi/Ff) x 100.

***RNA sequencing for the mice and the human samples***: Mice not used for BAL underwent overdose of ketamine (500 mg/kg i.v.) and xylazine (50mg/kg i.v.), bilateral thoracotomy, and exsanguination by right ventricular puncture. The whole lung was placed in RNA Shield (Zymo research, Irvine, CA), incubated at 4℃ overnight, and then frozen at -20℃. Frozen lungs were thawed, minced, homogenized, and then samples extracted using Quick-RNA mini prep plus kit (Zymo research, Irvine, CA). In humans, tracheal aspirate samples were collected on the day of enrollment and stored at -80C in RNAse-free conditions [1]. NA was extracted using the Allprpep kit (Qiagen and then ribodepleted using FastSelect (Qiagen) before undergoing library preparation using the NEBNext Ultra II RNASeq Kit (New England Biolabs) and and paired-end Illumina sequencing on an Novaseq 6000. Human and mouse samples were sequenced separately.

***Statistical analysis:*** All data were tested for normality with Shapiro-Wilk tests. Comparisons of each group were made with one-way ANOVA followed by Tukey’s multiple comparisons tests for normally distributed parameters, or with Kruskal-Wallis test followed by Dunn’s multiple comparisons for skewed parameters. Two-way analysis of variance for repeated measures followed by Tukey’s multiple comparison test was used to evaluate the effect of each treatment on oxygenation, body temperature and percentage body weight change. In cases of missing data, a mixed-effects model was used (body temperature and percentage body weight change). All tests were two-tailed, and differences were considered to be statistically significant when p < 0.05. Statistical analysis and graph production were done with Prism (GraphPad, La Jolla, CA). Group size was determined to ensure adequate statistical power based on our extensive experience with the models of acute lung injury [4]. All data are presented as means ± standard deviation (SD).

***Bioinformatics analysis:*** Transcriptomic analysis was performed using established bioinformatics pipelines. Briefly,pairwise comparison of treatment groups was performed using *limma-voom* and pathway analysis was performed using *fgsea* to compare differentially expressed genes to the Reactome database. Weighted gene co-expression network analysis (WGCNA) was performed using *CEMitool*. Results were adjusted for multiple hypothesis testing using the Benjamini-Hochberg method, and an adjusted p-value less than 0.1 was considered to be statistically significant.

Sequencing reads were pseudoaligned with *kallisto* to the human (ENSEMBL v. 99) or murine genomes (GCA_000001635.9) and gene-level counts were generated using *tximport*. Sample quality was assessed by principal components analysis and hierarchical clustering. One murine sample was discarded as a technical outlier. Differential expression was performed using the *voomLmFit* function from the *edgeR* package to fit a linear model of *voom*-transformed gene expression against experimental condition for mice or observational group for humans. Linear models were adjusted for experimental and sequencing batch effects. Moderated *t*-statistics and log-fold differences in gene expression were calculated using robust empirical Bayesian statistics. Gene set enrichment analysis (GSEA) was performed using *fgsea*. Reference gene lists from the Reactome database were downloaded using *msigdbr,* which also maps murine gene orthologs to human gene lists. Weighted gene co-expression network analysis (WGCNA) was performed using *CEMiTool* to analyze batch-corrected count data from the human observational cohort after applying the variance-stabilizing transformation. Batch correction for WGCNA analysis was performed using the *ComBat_seq* function from the *sva* package. Results were adjusted for multiple hypothesis testing using the Benjamini-Hochberg method, and an adjusted p-value less than 0.1 was considered to be statistically significant.

**Apendix-2. SUPPLEMENTAL RESULTS**

To further characterize gene expression patterns humans and mice with pneumonia, we used the *CEMiTool* package to perform an integrated co-expression analysis. *CEMiTool* determines the optimal settings for network analysis and then identifies modules of co-expressed genes using WGCNA. *CEMiTool* then tests for overrepresentation of annotated gene sets in gene expression modules present in the data, and merges gene expression modules with interactome data to identify candidate hubs for each network.

*CEMiTool* identified 12 gene expression modules in human tracheal aspirate transcriptomes. GSEA of module genes found reproducible enrichment of six modules in humans and mice with *S. pneumoniae* pneumonia, while three modules had reproducible enrichment with the addition of corticosteroid therapy *(****Supplementary Figure S1A***). Integration of these gene modules with interactome data identified central genes in each module with known roles in the pathogenesis of sepsis and ARDS (***Supplementary Figure S1B***). For example, *CXCR2*, a known mediator of neutrophilic alveolar inflammation, was a hub in Module 1; *CLEC4D* and *S100A8,* which are elevated in neutrophils from the SRS1 sepsis endotype [9], were key nodes in Module 4; and markers of cytotoxic T cells, including *CD3E* and *GZMB*, were key nodes in Module 12. Steroids increased the expression of Module 9, which included prostaglandin D_2_ synthase (PTGDS) as a central hub, in humans and in mice. Glucocorticoids induce the expression of *PTGDS* *[10]*, and prostaglandin D_2_ has pro-resolution effects in lung injury models [11]. Overrepresentation analysis of Reactome pathways in each module can be found in ***Supplementary Figure S2*.**

**Apendix-3. SUPPLEMENTAL FIGURE LEGENDS**

**Figure S1.** (*A*) Body temperature and body weight were measured 12, 20, 32 hours after bacterial inoculation. Infected mice became hypothermic at 12 hours after infection. Regardless of dexamethasone treatment, ceftriaxone attenuated hypothermia 32 hours after infection. n = 11-12/group. (*B*) Body weight (BW) loss was larger in the infected mice. There was no overall significant difference in body weight change among the treatment groups. n = 11-12/group.

All data are shown as means ± SD. Statistical differences between groups were calculated with two-way repeated ANOVA followed by Tukey’s multiple comparison test.

*P < 0.05 compared with control.

^†^P < 0.05 compared with groups without ceftriaxone.

**Figure S2**. (*A*) The number of neutrophils in bronchoalveolar lavage (BAL) fluid was elevated in the infected mice. The combination of dexamethasone and ceftriaxone reduced the number of neutrophils compared with untreated and ceftriaxone alone. n = 7-8/group. (*B*) The number of macrophages in BAL was not significantly different among all the groups. n = 7-8/group.

All data are shown as means ± SD. Statistical differences between groups were calculated with one-way ANOVA followed by Tukey’s multiple comparison test.

*P < 0.05, **P < 0.01, ****P < 0.0001.

**Figure S3.** (*A*) RAGE, a type I epithelial cell injury marker was measured in bronchoalveolar lavage (BAL) fluid. There was a significant increase in RAGE in pneumococcus-infected mice, indicating the alveolar cell injury. There was no statistically significant difference between untreated controls and other treatment groups. n = 7-8/group. (*B*) Alveolar fluid clearance was measured 36 hours after infection. Alveolar fluid clearance was substantially impaired with pneumococcal pneumonia. There was no statistically significant difference between untreated controls and other treatment groups, suggesting that neither dexamethasone nor ceftriaxone enhanced alveolar fluid clearance in the experimental period. n = 9-14/group.

All data are shown as means ± SD. Statistical differences between groups were calculated with one-way ANOVA followed by Tukey’s multiple comparison test.

*P < 0.05, **P < 0.01, ****P < 0.0001.

**Figure S4.** Biomarkers including IL-10, IL-2, IP-10, IL-4, IL-5, IL-22, IL-9, IL-13, IL-27, IL-23, IL-12p70, GM-CSF, RANTES, MIP-1α, MCP-3, IL-17A, MIP-2α, Eotaxin, IL-18, and MIP-1β were measured. n = 7-8/group.

All data are shown as means ± SD. Statistical differences between groups were calculated with one-way ANOVA followed by Tukey’s multiple comparison test or Kruskal-Wallis followed by Dunn’s multiple comparison.

*P < 0.05, **P < 0.01, ***P < 0.001, ****P < 0.0001.

^†^P < 0.05 compared with infected groups.

**Figure S5.** (*A*) Gene networks for modules identified by weighted gene co-expression network analysis (WGCNA) using interactome data from CEMiTool. Labeled genes are the ten most connected hubs in each network. (*B*) Dot plot of gene set enrichment analysis (GSEA) net enrichment scores (NES) for WGCNA modules. Red indicates genes in the Reactome pathway are relatively more highly expressed in humans or mice with *S. pneumoniae* treated with antibiotics in first and second rows, and more highly expressed in humans or mice who received steroids in the third and fourth rows. A solid circle indicates GSEA FDR <0.1.

**Figure S6.** Overrepresentation analysis (ORA) of gene modules identified by weighted gene co-expression network analysis (WGCNA). Bar plots show pathways from the Reactome database on the y-axis and -log_10_ adjusted p-value for overlap between genes in the module and genes in Reactome pathways on the x-axis. The dashed vertical line identifies an FDR < 0.1.

**Supplementary Data 1:** RNASeq gene counts for (*A*) human subject tracheal aspirates and (*B*) mouse whole lung homogenate. Counts for each gene, identified by Ensembl gene ID, are in each row, and data from each subject/mouse is in a separate column.

**Supplementary Data 2:** Differential expression results from *limma* for (*A*) SP with antibiotics vs. controls in human subjects, (*B*) SP with antibiotics and steroids vs. SP with antibiotics in human tracheal aspirates, (*C*) SP plus ceftriaxone vs. controls in mice, (*D*) SP plus ceftriaxone and dexamethasone vs. SP with ceftriaxone in mice.

**Supplementary Data 3:** Gene set enrichment analysis results for differential gene expression for (*A*) SP with antibiotics vs. controls in human subjects, (*B*) SP with antibiotics and steroids vs. SP with antibiotics in human tracheal aspirates, (*C*) SP plus ceftriaxone vs. controls in mice, (*D*) SP plus ceftriaxone and dexamethasone vs. SP with ceftriaxone in mice. ES: Enrichment score, NES: Net enrichment score.

**Supplementary Data 4:** Module assignments for genes from weighted gene co-expression network analysis.

**Apendix-4. SUPPLEMENTAL TABLE**

**S-Table 1 Characteristics of patients included in this study**

|  | Overall | Control | SP | | * | † |
| --- | --- | --- | --- | --- | --- | --- |
|  |  |  | No Steroids | Steroids |  |  |
| N | 15 | 5 | 5 | 5 |  |  |
| ARDS | 6 (40) | 0 (0) | 3 (60) | 3 (60) | 0.168 | 1.00 |
| *S. pneumoniae* culture |  | - | 2 (40) | 3 (60) | 0.429 | 1.00 |
| Age (years) | 56 (20) | 66 (23) | 42 (12) | 59 (19) | 0.077 | 0.131 |
| Female | 3 (20) | 3 (60) | 0 (0) | 0 (0) | 0.168 | NA |
| BMI (kg/m^2^) | 27 (10) | 26 (5) | 28 (15) | 27 (8) | 0.768 | 0.908 |
| PF ratio (mmHg) | 196 (107) | 298 (90) | 184 (97) | 127 (73) | 0.114 | 0.332 |
| Vasopressors | 7 (47) | 1 (20) | 1 (20) | 5 (100) | 1.00 | 0.053 |
| SOFA | 9 (6) | 5 (2) | 8 (5) | 14 (5) | 0.336 | 0.071 |
| APACHE II | 25 (12) | 18 (6) | 21 (11) | 36 (9) | 0.951 | 0.046 |
| VFD | 16 (10) | - | 23 (4) | 12 (11) |  | 0.290 |

Data are presented as mean (SD) for continuous variables, and as N (%) for categorical variables. Continuous variables were compared using *t*-tests and categorical variables were compared using Fisher’s exact test.

***Abbreviations***: BMI: Body mass index; PF ratio: Arterial oxygen tension (P_a_O_2_) to fraction of inspired oxygen (F_i_O_2_) ratio; SOFA: Sequential Organ Failure Assessment score; APACHE: Acute Physiology and Chronic Health Evaluation score; VFD: Ventilator free days

*p-value for comparisons between patients with *S. pneumoniae* who did not receive steroids and controls

^†^p-value for comparisons between patients with *S. pneumoniae* who received steroids and those who did not receive steroids.

**REFERENCES**

1. Sarma A, Christenson SA, Byrne A, Mick E, Pisco AO, DeVoe C, Deiss T, Ghale R, Zha BS, Tsitsiklis A *et al*: **Tracheal aspirate RNA sequencing identifies distinct immunological features of COVID-19 ARDS**. *Nature Communications* 2021, **12**(1):5152.

2. Langelier C, Kalantar KL, Moazed F, Wilson MR, Crawford ED, Deiss T, Belzer A, Bolourchi S, Caldera S, Fung M *et al*: **Integrating host response and unbiased microbe detection for lower respiratory tract infection diagnosis in critically ill adults**. *Proc Natl Acad Sci U S A* 2018, **115**(52):E12353-e12362.

3. Su X, Lee JW, Matthay ZA, Mednick G, Uchida T, Fang X, Gupta N, Matthay MA: **Activation of the alpha7 nAChR reduces acid-induced acute lung injury in mice and rats**. *American journal of respiratory cell and molecular biology* 2007, **37**(2):186-192.

4. Gotts JE, Bernard O, Chun L, Croze RH, Ross JT, Nesseler N, Wu X, Abbott J, Fang X, Calfee CS *et al*: **Clinically relevant model of pneumococcal pneumonia, ARDS, and nonpulmonary organ dysfunction in mice**. *American journal of physiology Lung cellular and molecular physiology* 2019, **317**(5):L717-L736.

5. Matute-Bello G, Downey G, Moore BB, Groshong SD, Matthay MA, Slutsky AS, Kuebler WM: **An official American Thoracic Society workshop report: features and measurements of experimental acute lung injury in animals**. *Am J Respir Cell Mol Biol* 2011, **44**(5):725-738.

6. Uchida T, Shirasawa M, Ware LB, Kojima K, Hata Y, Makita K, Mednick G, Matthay ZA, Matthay MA: **Receptor for advanced glycation end-products is a marker of type I cell injury in acute lung injury**. *American journal of respiratory and critical care medicine* 2006, **173**(9):1008-1015.

7. Fukuda N, Folkesson HG, Matthay MA: **Relationship of interstitial fluid volume to alveolar fluid clearance in mice: ventilated vs. in situ studies**. *Journal of applied physiology (Bethesda, Md : 1985)* 2000, **89**(2):672-679.

8. Flodby P, Kim YH, Beard LL, Gao D, Ji Y, Kage H, Liebler JM, Minoo P, Kim KJ, Borok Z *et al*: **Knockout Mice Reveal a Major Role for Alveolar Epithelial Type I Cells in Alveolar Fluid Clearance**. *Am J Respir Cell Mol Biol* 2016, **55**(3):395-406.

9. Kwok AJ, Allcock A, Ferreira RC, Cano-Gamez E, Smee M, Burnham KL, Zurke Y-X, Novak A, Darwent M, Baron T *et al*: **Neutrophils and emergency granulopoiesis drive immune suppression and an extreme response endotype during sepsis**. *Nature Immunology* 2023, **24**(5):767-779.

10. Tokudome S, Sano M, Shinmura K, Matsuhashi T, Morizane S, Moriyama H, Tamaki K, Hayashida K, Nakanishi H, Yoshikawa N *et al*: **Glucocorticoid protects rodent hearts from ischemia/reperfusion injury by activating lipocalin-type prostaglandin D synthase–derived PGD2 biosynthesis**. *The Journal of clinical investigation* 2009, **119**(6):1477-1488.

11. Murata T, Aritake K, Tsubosaka Y, Maruyama T, Nakagawa T, Hori M, Hirai H, Nakamura M, Narumiya S, Urade Y *et al*: **Anti-inflammatory role of PGD2 in acute lung inflammation and therapeutic application of its signal enhancement**. *Proc Natl Acad Sci U S A* 2013, **110**(13):5205-5210.
